# From Cardiovascular-Kidney-Metabolic Syndrome to Cardiovascular-Renal-Hepatic-Metabolic Syndrome: Proposing an Expanded Framework

**DOI:** 10.3390/biom15020213

**Published:** 2025-02-02

**Authors:** Nikolaos Theodorakis, Maria Nikolaou

**Affiliations:** 1 NT-CardioMetabolics, Clinic for Metabolism and Athletic Performance, 47 Tirteou Str., 17564 Palaio Faliro, Greece; 2 Department of Cardiology & Preventive Cardiology Outpatient Clinic, Amalia Fleming General Hospital, 14, 25th Martiou Str., 15127 Melissia, Greece; m.nikolaou@flemig-hospital.gr; 3 School of Medicine, National and Kapodistrian University of Athens, 75 Mikras Asias, 11527 Athens, Greece

**Keywords:** cardiometabolic medicine, arterial hypertension, dyslipidemia, diabetes mellitus, obesity, heart failure, chronic kidney disease, metabolic dysfunction-associated steatotic liver disease, cardiovascular-kidney-metabolic syndrome, cardiovascular-renal-hepatic-metabolic syndrome

## Abstract

Cardiometabolic diseases represent an escalating global health crisis, slowing or even reversing earlier declines in cardiovascular disease (CVD) mortality. Traditionally, conditions such as obesity, type 2 diabetes mellitus (T2DM), atherosclerotic CVD, heart failure (HF), chronic kidney disease (CKD), and metabolic dysfunction-associated steatotic liver disease (MASLD) were managed in isolation. However, emerging evidence reveals that these disorders share overlapping pathophysiological mechanisms and treatment strategies. In 2023, the American Heart Association proposed the Cardiovascular-Kidney-Metabolic (CKM) syndrome, recognizing the interconnected roles of the heart, kidneys, and metabolic system. Yet, this model omits the liver—a critical organ impacted by metabolic dysfunction. MASLD, which can progress to metabolic dysfunction-associated steatohepatitis (MASH), is closely tied to insulin resistance and obesity, contributing directly to cardiovascular and renal impairment. Notably, MASLD is bidirectionally associated with the development and progression of CKM syndrome. As a result, we introduce an expanded framework—the Cardiovascular-Renal-Hepatic-Metabolic (CRHM) syndrome—to more comprehensively capture the broader inter-organ dynamics. We provide guidance for an integrated diagnostic approach aimed at halting progression to advanced stages and preventing further organ damage. In addition, we highlight advances in medical management that target shared pathophysiological pathways, offering benefits across multiple organ systems. Viewing these conditions as an integrated whole, rather than as discrete entities, and incorporating the liver into this framework fosters a more holistic management strategy and offers a promising path to addressing the cardiometabolic pandemic.

## 1. Introduction

We are witnessing a global pandemic of cardiometabolic disorders, which has slowed or even reversed earlier declines in cardiovascular disease (CVD) mortality, prompting stronger efforts to improve prevention and treatment strategies [1]. Over the past five years, our understanding of these conditions has evolved dramatically. Obesity, type 2 diabetes mellitus (T2DM), atherosclerotic CVD (ASCVD), heart failure (HF), chronic kidney disease (CKD), and metabolic dysfunction-associated steatotic liver disease (MASLD) were once treated as separate conditions. Today, they are recognized as interconnected disorders sharing common pathophysiological pathways that drive parallel disease progression [2]. Simultaneously, new medications such as sodium-glucose cotransporter 2 inhibitors (SGLT2i), glucagon-like peptide-1 receptor agonists (GLP-1RAs), combined gastric inhibitory polypeptide (GIP)/GLP-1 receptor agonists, and finerenone have demonstrated benefits across multiple conditions in this spectrum, improving both quality of life and hard clinical outcomes [3,4].

In 2023, the American Heart Association (AHA) introduced the Cardiovascular-Kidney-Metabolic (CKM) syndrome to emphasize the interconnected roles of the heart, blood vessels, kidneys, and metabolism. However, this model does not fully account for the liver’s role within this spectrum. MASLD, the hepatic manifestation of metabolic dysregulation, is both a driver and a consequence of metabolic dysfunction. Its progression to metabolic dysfunction-associated steatohepatitis (MASH) is closely intertwined with insulin resistance and obesity, creating a bidirectional relationship that exacerbates the development and progression of atherosclerosis, HF, and CKD [5,6].

**Figure 1:**
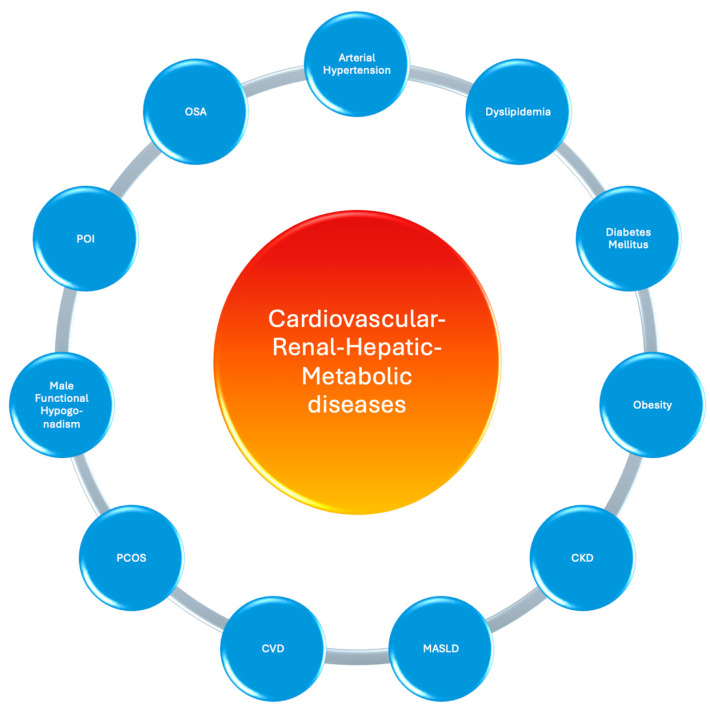
The spectrum of Cardiovascular-Renal-Hepatic-Metabolic diseases. Abbreviations. ASCVD (atherosclerotic cardiovascular disease); CKD (chronic kidney disease); CVD (cardiovascular disease); HF (heart failure); MASLD (metabolic dysfunction-associated steatotic liver disease); OSA (obstructive sleep apnea); PCOS (polycystic ovarian syndrome); POI (primary ovarian insufficiency).

According to the 2024 guidelines of the EASL-EASD-EASO, cardiometabolic criteria are now part of the definition and diagnosis of MASLD, which was previously termed non-alcoholic fatty liver disease (NAFLD) before 2023 [7]. Specifically, the most common cardiometabolic disorders associated with MASLD include overweight/obesity (∼90%), prediabetes/T2DM (∼60%), prehypertension/arterial hypertension (∼85%), hypertriglyceridemia (∼75%), low high-density lipoprotein cholesterol (HDL-C, ∼60%), and HF with preserved ejection fraction (HFpEF, ∼50%) [8,9]. Furthermore, individuals with MASLD are at an increased risk of cardiovascular mortality (hazard ratio [HR] 1.30), non-fatal CVD (HR 1.40), coronary artery disease (odds ratio [OR] 1.33), HF (OR 1.5), CKD (HR 1.43), T2DM, and diabetes-related peripheral polyneuropathy (HRs 2.19 and 2.48, respectively), and obstructive sleep apnea (OSA, HR 2.22) [7]. This group of associated conditions and additional disorders related to increased cardiometabolic risk create the spectrum of Cardiovascular-Renal-Hepatic-Metabolic diseases (Figure 1).

Current evidence highlights that MASLD is not only common among individuals with other cardiometabolic diseases, but also plays a key role in the progression of CKM syndrome. This underscores the need for a more comprehensive framework that includes MASLD and its intricate connections to cardiometabolic and renal health. In this context, we propose expanding the CKM syndrome to a Cardiovascular-Renal-Hepatic-Metabolic (CRHM) syndrome. This new framework recognizes the liver’s central role in inter-organ dynamics and aims to improve clinical strategies for prevention, diagnosis, and management. By defining this expanded syndrome, exploring its interconnected pathways, and outlining integrated diagnostic and treatment approaches, this manuscript hopes to provide a more holistic and effective way to address these overlapping diseases during the ongoing cardiometabolic health crisis.

## 2. Defining and Staging the CRHM Syndrome

The concept of metabolic syndrome (MetS) has long been recognized in medical practice, with insulin resistance identified as a key pathophysiological driver. MetS is clinically defined by the presence of three or more of the following five criteria [5,6]:

▪ Waist circumference ≥88 cm for women and ≥102 cm for men (≥80 cm for women and ≥90 cm for men in individuals of Asian descent).
▪ HDL-C <40 mg/dL for men and <50 mg/dL for women.
▪ Triglycerides (TG) ≥150 mg/dL.
▪ Elevated blood pressure (systolic ≥130 mm Hg or diastolic ≥80 mm Hg, or use of antihypertensive medications).
▪ Fasting blood glucose ≥100 mg/dL, or the use of antidiabetic medications.

Building on the foundation of MetS, in 2023, the AHA introduced the CKM syndrome, which has been defined as “A systemic disorder characterized by pathophysiological interactions among metabolic risk factors, CKD, and the cardiovascular system leading to multiorgan dysfunction and a high rate of adverse cardiovascular outcomes. CKM syndrome includes both individuals at risk for CVD due to the presence of metabolic risk factors, CKD, or both and individuals with existing CVD that is potentially related to or complicates metabolic risk factors or CKD.”

Although this model addresses the interplay between the cardiovascular, renal, and metabolic systems, it does not fully capture the role of the liver. MASLD, and particularly its progression to MASH and cirrhosis, is bidirectionally associated with metabolic dysregulation, atherosclerosis, HF, and CKD. With this in mind, we propose expanding the CKM syndrome to a Cardiovascular-Renal-Hepatic-Metabolic (CRHM) syndrome, defined as: “A systemic disorder that leads to parallel multiorgan dysfunction driven by shared pathophysiological mechanisms, including metabolic inflammation (meta-inflammation) and dysregulation, especially insulin resistance. This syndrome includes early stages with risk factors—where prevention can slow progression—as well as established cardiovascular, renal, and hepatic manifestations and the interactions between them, including ASCVD, HF, CKD, and MASLD/MASH/cirrhosis.” Based on this definition, we propose a modified staging system that integrates the liver as a central player (Table 1). By integrating the liver into this framework, clinicians and researchers can adopt a more comprehensive view of how metabolic, cardiovascular, renal, and hepatic factors interact. This lays the groundwork for a more cohesive approach to diagnosis, risk stratification, and therapeutic interventions.

Our proposed framework aligns closely with the recent concept of classifying obesity into preclinical and clinical stages. On January 14, 2025, *The Lancet Diabetes & Endocrinology* published its commission on the definition and diagnostic criteria of clinical obesity [10]. The commission defines obesity as a condition characterized by excess adiposity, with or without abnormal adipose tissue distribution or function, and with multifactorial causes that remain incompletely understood. Preclinical obesity is described as a state of excess adiposity without current evidence of organ dysfunction or limitations in daily activities. However, it carries a variable, but generally heightened, risk of progressing to clinical obesity and developing related non-communicable diseases, such as type 2 diabetes, cardiovascular disease, certain cancers, and mental health disorders. In contrast, clinical obesity is defined as a chronic, systemic illness associated with evidence of impaired organ or tissue function and/or age-adjusted limitations in daily activities. These limitations may reflect the specific impact of excess adiposity on mobility and basic activities of daily living, such as bathing, dressing, toileting, continence, and eating. In our proposed framework, Stage 1 corresponds to preclinical obesity, representing early dysfunction due to adiposity but without significant clinical manifestations. Stages 2 through 4 correspond to clinical obesity, capturing the progressive systemic effects of excess adiposity on organ function.

## 3. Pathophysiology of the CRHM Syndrome

### 3.1. Excess Adiposity

The pathophysiology of the CRHM syndrome is grounded by how excess/dysfunctional adiposity and additional cardiometabolic risk factors are bidirectionally associated with the development of renal, hepatic, and cardiovascular dysfunction. The Central driver of this syndrome process is excess adiposity, which is characterized by chronic inflammation and drives the development of other CRHM risk factors, including arterial hypertension, T2DM, and dyslipidemia, while also promoting atherosclerosis, cardiac, renal and hepatic dysfunction. Understanding these pathways helps clinicians recognize that what begins with adipose dysfunction and mild metabolic disturbances can escalate into advanced HF, CKD, and liver cirrhosis.

#### 3.1.1. Adiposity and Insulin Resistance

Excess and especially dysfunctional adipose tissue, especially visceral, is characterized by increased infiltration of immune cells, particularly M1 pro-inflammatory macrophages, and reduced angiogenesis. These changes create hypoxia within adipose depots, which further exacerbates local inflammation. Pro-inflammatory cytokines such as tumor necrosis factor-α (TNF-α), interleukin (IL)-6, and IL-1β are released by macrophages and dysfunctional adipocytes. The interleukin inflammation resulting from this metabolic dysregulation is referred to as “meta-inflammation,” which disrupts normal insulin signaling in peripheral tissues, including muscle, liver, and adipose tissue. Specifically, pro-inflammatory cytokines activate intracellular kinases, such as JNK and IKK-β, which phosphorylate insulin receptor substrate (IRS) proteins on serine residues instead of tyrosine. This aberrant phosphorylation impairs downstream signaling, reducing glucose uptake in peripheral tissues like skeletal muscle and adipose tissue while increasing hepatic gluconeogenesis [11,12].

Moreover, enlarged and dysfunctional adipose tissue releases higher levels of free fatty acids (FFAs) into circulation. These FFAs accumulate in liver and muscle cells, causing lipotoxicity. This ectopic fat deposition directly impairs insulin signaling and mitochondrial function, further exacerbating metabolic dysfunction [11].

Another hallmark of obesity is the dysregulation of adipokines. In obesity, leptin—a hormone responsible for regulating satiety and energy balance—is chronically overproduced, leading to leptin resistance. This state reduces the brain’s ability to regulate appetite and energy expenditure, further driving weight gain. Leptin resistance is also closely linked to the development of insulin resistance. In addition, resistin, an adipokine that is elevated in obesity, promotes meta-inflammation and exacerbates insulin resistance. Conversely, adiponectin, an insulin-sensitizing and anti-inflammatory adipokine, decreases as adiposity increases [13].

#### 3.1.2. Adiposity and Dyslipidemia

Excess adipose tissue continuously releases FFAs into the portal and systemic circulation. The liver utilizes these FFAs to synthesize TGs, which are then packaged into very low-density lipoproteins (VLDLs). Overproduction of VLDLs elevates plasma TG levels, a hallmark of obesity-related dyslipidemia. High levels of triglyceride-rich VLDLs promote the activity of cholesteryl ester transfer protein (CETP), which facilitates the exchange of triglycerides from VLDLs with cholesteryl esters from HDL. This process depletes HDL particles, resulting in lower plasma HDL levels. Moreover, the increased presence of VLDLs contributes to the formation of small dense low-density lipoprotein (sdLDL) particles, which are more atherogenic. Meta-inflammation, dysregulated adipokine profiles, and insulin resistance further disrupt lipoprotein metabolism, compounding the dyslipidemic profile associated with obesity [14].

#### 3.1.3. Adiposity and Arterial Hypertension

Meta-inflammation and dysregulated adipokine profiles in obesity can activate the sympathetic nervous system (SNS) and the renin-angiotensin-aldosterone system (RAAS), contributing to endothelial dysfunction and reduced nitric oxide (NO) production. These changes result in an increased heart rate and peripheral vascular resistance, as well as elevated adrenal aldosterone secretion, ultimately leading to elevated blood pressure. Chronic activation of the RAAS and SNS also promotes smooth muscle hypertrophy in blood vessels, further increasing peripheral vascular resistance. Additionally, dysfunctional adipose tissue directly stimulates aldosterone secretion, which contributes to sodium retention and exacerbates hypertension [15].

#### 3.1.4. Adiposity and Renal Dysfunction

In the early stages of obesity and insulin resistance, increased blood volume and cardiac output (CO) can elevate the glomerular filtration rate (GFR), a phenomenon known as “hyperfiltration.” This hyperfiltration leads to chronic glomerular hypertension and dysfunction. Additionally, neurohormonal activation increases efferent arteriolar tone, raising intraglomerular pressure. The elevated mechanical stress within the glomeruli can damage podocytes, which are key cells in the kidney’s filtration barrier, resulting in proteinuria and progressive nephron loss. Furthermore, chronic inflammation can contribute to renal injury by directly damaging tubular cells and promoting fibrosis [16].

#### 3.1.5. Adiposity, MASLD and HFpEF

Excess FFAs released from adipose tissue accumulate inside cardiomyocytes and hepatocytes, causing steatosis. Insulin resistance impairs the hepatic and myocardial ability to efficiently utilize TGs and oxidize FFAs, perpetuating the intracellular buildup of lipid droplets. Excess FFAs are prone to oxidation, generating reactive oxygen species (ROS) that lead to myocardial and hepatic damage and dysfunction. Furthermore, dysfunctional adiposity drives meta-inflammation and the release of pro-inflammatory cytokines, stimulating fibrotic remodeling and left ventricular hypertrophy—hallmarks of HFpEF—as well as liver fibrosis, which is a hallmark of MASLD. Dysregulated adipokine profiles, increased aldosterone production, and RAAS activation, resulting from dysfunctional adiposity, further perpetuate myocardial and hepatic damage and fibrosis. Over time, these processes may progress from asymptomatic myocardial dysfunction to HFpEF, as well as from MASLD to MASH and liver cirrhosis [17,18]. Reflecting their shared pathophysiology, HFpEF has been termed the “NASH of the heart,” underscoring the parallel roles of adiposity-driven toxicity and meta-inflammation in these two metabolically driven diseases [19].

### 3.2. Coexistence of Diabetes and Hypertension and Progression to CKD

When diabetes and hypertension coexist, their deleterious effects on the kidneys are significantly amplified:

▪ Glomerular Hyperfiltration: Both conditions elevate glomerular pressure and exacerbate hyperfiltration, accelerating damage to podocytes and other glomerular cells. In diabetes, chronic hyperglycemia increases glucose filtration through the kidneys. To compensate, renal tubules reabsorb more glucose and sodium, raising intraglomerular pressure and promoting hyperfiltration. Similarly, systemic hypertension transmits high pressure to the glomeruli, leading to hyperfiltration in early stages and eventually progressing to glomerular sclerosis (hardening and scarring) [20,21].
▪ RAAS: Insulin resistance and hyperglycemia can enhance RAAS activity, which is often elevated in idiopathic arterial hypertension. Elevated angiotensin II causes preferential constriction of the efferent arteriole, increasing intraglomerular pressure. Increased aldosterone secretion and sodium and fluid retention further compound this pressure, leading to chronic glomerular injury. Over time, RAAS-driven damage contributes to structural changes in renal vasculature, such as hyaline arteriosclerosis in afferent arterioles and hyperplastic arteriosclerosis in more severe cases [20,21].
▪ Vascular Remodeling: Persistent hypertension thickens the walls of small renal arteries and arterioles, narrowing the lumen and impairing blood flow. This ischemic injury to nephrons exacerbates kidney damage [20,21].
▪ Advanced Glycation End Products (AGEs): Prolonged hyperglycemia promotes the formation of AGEs in kidney tissues. These AGEs alter protein structures, such as collagen in the glomerular basement membrane, triggering inflammation, fibrosis, and oxidative stress [20,21].
▪ Endothelial Dysfunction and Chronic Inflammation: Endothelial dysfunction, driven by pro-inflammatory cytokines and oxidative stress from both diabetes and hypertension, damages glomeruli and renal vessels. Chronic inflammation activates mesangial cells and fibroblasts, leading to extracellular matrix deposition, nephron loss, and a gradual decline in GFR [20,21].

### 3.3. Liver-Cardiovascular Interactions

#### 3.3.1. How Cardiac Dysfunction Affects the Liver

▪ Reduced Hepatic Perfusion: In cardiac dysfunction, particularly HF with reduced ejection fraction (HFrEF), decreased CO leads to diminished hepatic perfusion. This inadequate perfusion limits nutrient and oxygen delivery to the liver, causing metabolic stress to the hepatocytes and contributing to hepatocellular injury [22].
▪ Congestive Hepatopathy: In HF, elevated central venous pressure is transmitted to the hepatic veins, resulting in sinusoidal congestion, a condition often referred to as congestive hepatopathy. HF also activates the RAAS, promoting sodium and water retention, further exacerbating hepatic congestion. Persistent congestion impairs hepatic microcirculation, diminishing oxygen and nutrient delivery to hepatocytes. The combination of sinusoidal congestion and low-grade inflammation activates hepatic stellate cells, leading to collagen production and extracellular matrix deposition. Over time, this process can contribute to ascites development and even progress to significant fibrosis and “cardiac cirrhosis” [23].
▪ Neurohormonal Activation: Cardiac dysfunction, particularly HFrEF, and to a lesser extent HFpEF, are associated with neurohormonal activation (activation of the SNS and RAAS), which increases systemic and splanchnic vascular resistance. While cirrhosis is often associated with low systemic vascular resistance, HF-driven RAAS activation can disrupt this balance, worsening portal hypertension and ascites. Aldosterone and angiotensin II contribute to tissue fibrosis, potentially exacerbating liver fibrosis in patients with ongoing liver injury [24,25].
▪ Chronic Inflammation and Oxidative Stress: ASCVD and HF are associated with elevated levels of pro-inflammatory cytokines (e.g., TNF-α, IL-6) and oxidative stress markers. These mediators can exacerbate underlying liver disease and perpetuate the cycle of hepatic injury and fibrosis [26,27].

#### 3.3.2. How Hepatic Dysfunction Affects the Cardiovascular System

▪ Hyperdynamic Circulation: In advanced liver cirrhosis, excessive production of vasodilatory mediators like NO, particularly in the splanchnic circulation, leads to a hyperdynamic circulatory state characterized by reduced systemic vascular resistance. Although arterial blood pressure may drop, the heart compensates by increasing CO to maintain perfusion. Over time, this cardiac stress can result in cardiac remodeling, reduced contractility, and eventual HF—a condition often termed “cirrhotic cardiomyopathy” [28].
▪ Neurohormonal Activation: In cirrhosis or severe liver dysfunction, overproduction of vasodilatory mediators such as NO in the splanchnic circulation which, together with hypoalbuminemia and decreased oncotic pressure, leads to markedly reduced effective arterial blood volume. This triggers compensatory activation of the RAAS and the SNS. RAAS activation induces vasoconstriction and increases afterload, leading to chronic left ventricular pressure overload. This can contribute to or exacerbate left ventricular hypertrophy and myocardial fibrosis, contributing to the development of “cirrhotic cardiomyopathy”. Additionally, elevated aldosterone levels drive sodium and water retention, causing volume overload [24,25].
▪ Ascites: Tense ascites may compromise venous return and cardiac filling, worsening HF symptoms [29].
▪ Toxic Metabolite Accumulation: In liver failure, the liver cannot efficiently clear ammonia and other metabolic byproducts, resulting in their accumulation. These toxins can stimulate systemic inflammation, oxidative stress, increase the risk of arrhythmias, promote atherosclerosis, and contribute to the development of “cirrhotic cardiomyopathy” [30].
▪ Chronic Inflammation and Oxidative Stress: Chronic liver disease, such as in MASH or cirrhosis, activates Kupffer cells and other immune cells, resulting in the release of pro-inflammatory cytokines (e.g., TNF-α, IL-6) and oxidative stress. This can directly damage cardiomyocytes and endothelial cells, contributing to atherosclerosis and the development of “cirrhotic cardiomyopathy” [31].
▪ Promotion of Dyslipidemia and Insulin Resistance in MASLD: In patients with MASLD, factors including lipotoxicity and meta-inflammation result in dysregulated hepatic lipid metabolism and decreased hepatic insulin sensitivity, leading to dyslipidemia, as well as increased glycogenolysis and gluconeogenesis resulting in hyperglycemia, significant risk factors for atherosclerosis [31].

### 3.4. Cardiovascular-Kidney Interactions

#### 3.4.1. How Cardiovascular Dysfunction Affects the Kidneys

▪ Reduced Renal Perfusion: In cardiac dysfunction, particularly HFrEF, decreased CO leads to diminished renal perfusion. This reduction lowers the GFR and is a hallmark of the cardiorenal syndrome, while it can also cause chronic ischemic renal damage and atrophy [32].
▪ Congestive nephropathy: Elevated central venous pressure resulting from cardiac dysfunction transmits backward into the renal veins, resulting in renal congestion. HF also activates the RAAS, promoting sodium and water retention, further exacerbating renal congestion. High venous pressure reduces the transrenal pressure gradient, impairing glomerular filtration. Over time, this process can cause fibrosis and progressive nephron loss [32].
▪ Neurohormonal Activation: Cardiac dysfunction, particularly HFrEF, and in less extent HFpEF, are associated with neurohormonal activation (activation of the SNS and RAAS), which increases systemic and splanchnic vascular resistance. Chronic RAAS activation is associated with glomerular damage, resulting in proteinuria, fibrosis, and progressive nephron loss. Chronic SNS activation exacerbates renal vasoconstriction, further reducing GFR and promoting ischemic renal injury [25,32].
▪ Chronic Inflammation and Oxidative Stress: ASCVD and HF are associated with elevated levels of pro-inflammatory cytokines (e.g., TNF-α, IL-6) and oxidative stress markers. These mediators can directly damage renal tubular cells and blood vessels, contributing to renal dysfunction [26,33].

#### 3.4.2. How Renal Dysfunction Affects the Cardiovascular System

▪ Volume Overload and Neurohormonal Activation: As kidney function declines, reduced sodium delivery to the distal tubule stimulates renin release, triggering activation of the RAAS. RAAS activation induces vasoconstriction and increases afterload, leading to chronic left ventricular pressure overload. This can contribute to or exacerbate left ventricular hypertrophy and myocardial fibrosis, resulting in stiffened ventricular walls and promoting diastolic dysfunction. Additionally, elevated aldosterone levels drive sodium and water retention, causing volume overload and increased right ventricular preload. This volume overload may lead to interventricular interaction through the leftward displacement of the interventricular septum, reducing left ventricular preload and subsequently diminishing CO. Furthermore, excessive fluid retention can contribute to or worsen pulmonary edema [34].
▪ Uremic Toxin Accumulation: Uremic toxins, such as urea and indoxyl sulfate, accumulate in advanced CKD and can directly impair myocardial function. These toxins also impair endothelial function by reducing NO production, and promote vasoconstriction, inflammation, atherosclerosis, and arrhythmogenesis [35].
▪ Anemia of CKD: Anemia of CKD occurs because failing kidneys produce less erythropoietin (EPO), leading to normocytic anemia. Furthermore, the uremic toxin indoxyl sulfate increases apoptosis in red cells, further contributing to the anemia in CKD [36]. In response, the heart attempts to increase the CO to meet peripheral oxygen demands, which can elevate left ventricular filling pressures and cause dyspnea. Chronic hyperdynamic circulation due to anemia of CKD places stress on the heart, potentially leading to a decline in cardiac function. Additionally, severe anemia may reduce the myocardial oxygen supply, causing ischemia and further impairing cardiac function, potentially resulting in type 2 myocardial infarction [36,37].
▪ Dysregulated Mineral Metabolism: CKD disrupts mineral metabolism, promoting vascular calcification, arterial stiffness, and aortic calcification. This increases afterload, further straining the heart and exacerbating cardiac dysfunction, while it can also contribute to accelerated atherosclerosis and myocardial ischemia [38].
▪ Chronic Inflammation and Oxidative Stress: CKD is associated with elevated levels of pro-inflammatory cytokines (e.g., TNF-α, IL-6) and oxidative stress markers. Indoxyl sulfate, in particular, acts as a significant endotheliotoxin contributing to the development of cardiovascular disease in individuals with CKD. Within endothelial cells, it triggers oxidative stress, inflammation, and thrombosis, all of which play a central role in endothelial dysfunction, myocardial dysfunction, and atherosclerosis [39].

### 3.5. Liver-Kidney Interactions

#### 3.5.1. How Hepatic Dysfunction Affects the Kidneys

▪ Splanchnic Vasodilation and Hypoalbuminemia: In cirrhosis or severe liver dysfunction, overproduction of vasodilatory mediators such as NO in the splanchnic circulation which, together with hypoalbuminemia and decreased oncotic pressure, leads to markedly reduced effective arterial blood volume, which can progress to hepatorenal syndrome [40].
▪ Neurohormonal Activation: Reduced effective arterial blood volume above compensatory activation of the RAAS and the SNS. Chronic RAAS activation can lead to with glomerular damage, proteinuria, fibrosis, and progressive nephron loss. Chronic SNS activation exacerbates renal vasoconstriction, further reducing GFR and promoting ischemic renal injury [24].
▪ Ascites: Tense ascites can directly impair renal arterial blood supply and venous return, further compromising kidney function [41].
▪ Toxic Metabolite Accumulation: In liver failure, the liver cannot efficiently clear ammonia and other metabolic byproducts, resulting in their accumulation. These toxins contribute to endothelial dysfunction, worsening renal vasoconstriction and promoting systemic and renal inflammation, further impairing kidney function [31,42].
▪ Systemic Inflammation and Oxidative Stress: Chronic liver injury, such as MASH or cirrhosis, activates Kupffer cells and other immune cells, resulting in the release of pro-inflammatory cytokines (e.g., TNF-α, IL-6) and oxidative stress. These mediators can directly damage renal tubular cells and blood vessels, contributing to renal dysfunction [31].

#### 3.5.2. How Renal Dysfunction Affects the Liver

▪ Volume Overload: As kidney function declines, reduced sodium and water excretion promotes fluid retention. In patients with liver disease who are already prone to fluid overload (e.g., ascites, edema), this exacerbates portal hypertension and venous congestion. Increased intra-abdominal pressure from worsening ascites can impair hepatic venous outflow, further deteriorating liver function [43].
▪ Neurohormonal Activation: Persistent activation of the RAAS and SNS in CKD increases systemic and splanchnic vascular resistance. While cirrhosis is often associated with low systemic vascular resistance, kidney-driven RAAS activation can disrupt this balance, worsening portal hypertension and ascites. Aldosterone and angiotensin II contribute to tissue fibrosis, potentially exacerbating liver fibrosis in patients with ongoing liver injury [44].
▪ Uremic Toxin Accumulation: CKD leads to the accumulation of uremic toxins such as urea, indoxyl sulfate, and p-cresol sulfate. At high concentrations, these toxins induce systemic inflammation and oxidative stress, adding to the burden on a compromised liver and exacerbating hepatic encephalopathy. Uremic toxins also damage endothelial cells, reducing hepatic microcirculation efficiency and aggravating hepatic inflammation and fibrosis [45].
▪ Chronic Inflammation and Oxidative Stress: CKD is associated with elevated levels of pro-inflammatory cytokines (e.g., TNF-α, IL-6) and oxidative stress markers. These mediators can exacerbate underlying liver disease and perpetuate the cycle of hepatic injury and fibrosis [39].

### 3.6. Additional Modifying Factors in the Pathophysiology of CRHM Syndrome

In the sections above, we have emphasized how adiposity serves as the central driver and initial event in the development and progression of the CRHM syndrome. However, in addition to this, aging, sex hormone dynamics, environmental stressors, and genetic factors also play pivotal roles in the pathophysiology and progression of the syndrome. Aging is intrinsically linked to increased frailty, systemic low-grade inflammation (“inflammaging”), and mitochondrial dysfunction, all of which exacerbate metabolic, cardiovascular, renal, and hepatic dysfunction [ml-omics]. Frailty contributes to sarcopenic obesity, impaired glucose metabolism, and reduced physiological reserves, further compounding organ dysfunction [46,47,48]. 

Sex dimorphism, driven by differences in sex hormones, adds another layer of complexity. Estrogen, for example, exerts protective effects on cardiovascular, hepatic, and metabolic health, while its decline in postmenopausal women is associated with increased risks of visceral adiposity, dyslipidemia, and systemic inflammation [49]. Similarly, testosterone deficiency in men is linked to insulin resistance, reduced lean mass, and heightened cardiovascular risk [50].

Environmental stressors, including air pollution, smoking, physical inactivity, and chronic psychosocial stress, can further amplify CRHM progression through shared pathways such as oxidative stress, endothelial dysfunction, and chronic inflammation. Exposure to fine particulate matter (PM2.5) and environmental toxins has been shown to exacerbate atherosclerosis, kidney damage, and hepatic steatosis [51].

Finally, genetic factors and epigenetics also play a crucial role. It is well-documented that some individuals without obesity develop T2DM, while others with mild abdominal obesity do so, and some with severe, long-standing obesity remain free of T2DM. This variability in susceptibility highlights how adiposity’s effects are influenced by genetic predispositions and epigenetic modifications. Recognizing these additional factors provides a more comprehensive understanding of the interconnected pathways driving the CRHM syndrome.

**Figure 2:**
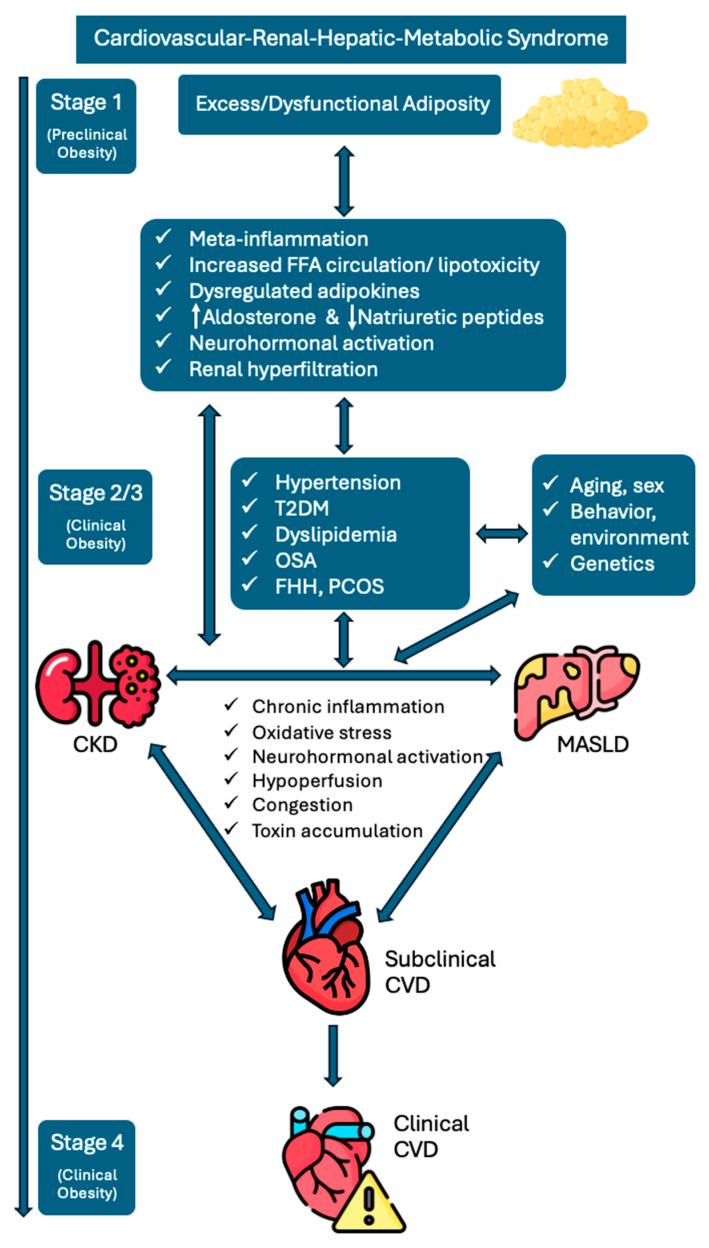
Schematic illustration of the pathophysiological interplay and progression of the CRHM syndrome. Adiposity marks the first stage of the CRHM syndrome, where excess or dysfunctional fat tissue triggers mechanisms like meta-inflammation and adipokine imbalance, driving CRHM risk factors such as hypertension, T2DM, and dyslipidemia. It also directly contributes to MASLD, CKD, and CVD. These conditions are interconnected through shared mechanisms, including neurohormonal activation, inflammation, oxidative stress, and congestion, leading to progressive multi-organ dysfunction. The final stage is established CVD, such as HF or ASCVD. Factors like age, sex, genetics, lifestyle, and environmental exposures further influence the development and progression of CRHM. Abbreviations. ASCVD (atherosclerotic cardiovascular disease); CKD (chronic kidney disease); CVD (cardiovascular disease); CRHM (cardiovascular-renal-hepatic-metabolic); FFA (free fatty acids); FHH (functional hypogonadotrophic hypogonadism); HF (heart failure); MASLD (metabolic dysfunction-associated steatotic liver disease); OSA (obstructive sleep apnea); PCOS (polycystic ovarian syndrome); T2DM (type 2 diabetes mellitus).

Figure 2 depicts the intricate pathophysiological interactions leading to the progression of the CRHM syndrome. In this framework, adiposity represents the first stage of the CRHM syndrome. Excess and/or dysfunctional adipose tissue, via a constellation of mechanisms (e.g., meta-inflammation, adipokine dysregulation), drives the development of additional CRHM risk factors (arterial hypertension, T2DM, and dyslipidemia) while also directly contributing to the pathogenesis and progression of MASLD, CKD, and CVD. These CRHM diseases are pathophysiologically interconnected through multiple mechanisms, including neurohormonal activation, chronic inflammation, oxidative stress, toxin accumulation, hypoperfusion, and congestion, perpetuating vicious cycles of progressive parallel organ dysfunction. The fourth and final stage of the CRHM syndrome is clinically established CVD, including HF and/or ASCVD. Demographic factors (e.g., age, sex, race), genetic predisposition, as well as environmental and lifestyle factors (e.g., smoking, alcohol, physical activity, nutrition, chronic stress, sleep, environmental pollution) are additional key mediators in the pathogenesis and progression of the CRHM syndrome.

## 4. Evaluation of the CRHM Syndrome

A comprehensive evaluation is crucial for identifying and characterizing the interplay of metabolic, cardiovascular, renal, and hepatic factors in individuals with the CRHM syndrome. One of the most important considerations in managing the CRHM syndrome is proper clinical staging, which allows for early interventions aimed at slowing or preventing progression to advanced stages. Below is a consolidated table (Table 2) that outlines primary assessments and possible follow-up investigations across multiple domains, along with the rationale for each. This structured approach aids clinicians in staging the disease, targeting early interventions, and preventing progression to advanced organ damage.

### Key Takeaways

▪ Multidimensional Screening: The CRHM syndrome spans various organ systems, and a thorough workup is necessary for recognizing early dysfunction. By evaluating adiposity, insulin resistance, blood pressure, lipid profile, renal function, hepatic health, and cardiovascular risk, clinicians can gain a complete picture of a patient’s metabolic and organ status.
▪ Risk Stratification and Staging: Each domain contributes to staging the CRHM syndrome. Early detection and classification allow for more targeted, timely interventions—whether they are lifestyle modifications, pharmacotherapies, or further investigations.
▪ Role of Specialized Tests: Secondary causes of obesity and hypertension, genetic testing for monogenic diabetes or familial hypercholesterolemia, advanced lipid testing (e.g., lipoprotein particle size, cholesterol efflux capacity), and advanced cardiovascular imaging studies may be indicated in complex or resistant cases. In patients with HF screening for multiple hormonal deficiency syndrome (growth hormone, testosterone, and/or triiodothyronine deficiency) could provide important additional insights [50,52,53,54]. Identifying these factors can significantly alter treatment strategies and improve outcomes.
▪ Patient-Centered Approach: Tailoring the diagnostic strategy to each patient’s clinical history, symptoms, and risk profile is essential. For instance, those with resistant hypertension warrant screening for primary hyperaldosteronism, whereas individuals with unusual patterns of dyslipidemia may need genetic testing for familial hypercholesterolemia.

By systematically integrating these evaluations, clinicians can build a robust framework for diagnosing, staging, and ultimately managing patients within the CRHM spectrum. This multifaceted approach enables early intervention and can slow or even halt progression toward advanced HF, renal failure, and/or cirrhosis, thereby improving long-term health outcomes.

## 5. Novel Therapeutic Options for the CRHM Syndrome

**Figure 3:**
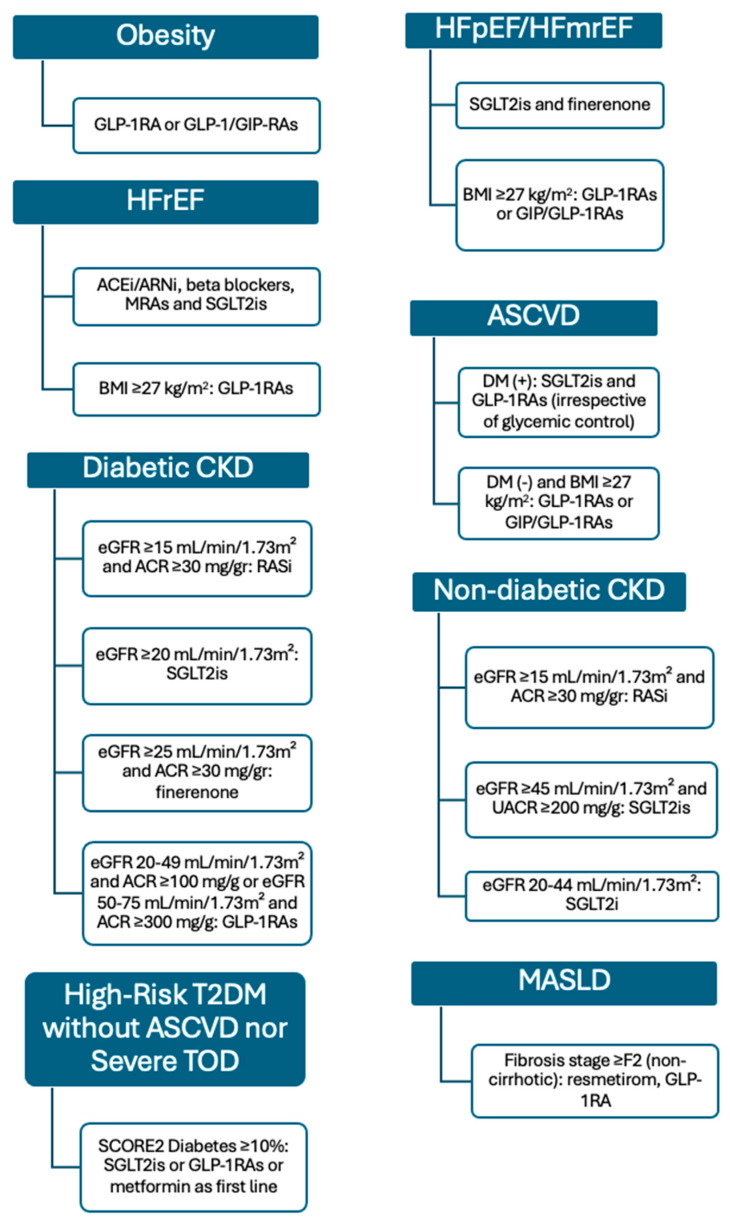
Novel and emerging therapeutic approaches for CRHM syndrome. We should note that the use of finerenone in HFpEF, as well as the use of GLP-1RAs and GIP/GLP-1RAs in HF; diabetic CKD and MASLD have not yet been recommended by guidelines because the evidence from trials is very recent. Abbreviations ACEi (angiotensin-converting enzyme inhibitors); ARNi (angiotensin receptor-neprilysin inhibitors); ASCVD (atherosclerotic cardiovascular disease); BMI (body mass index); CKD (chronic kidney disease); DM (diabetes mellitus); eGFR (estimated glomerular filtration rate); GIP (glucose-dependent insulinotropic polypeptide); GLP-1RA (glucagon-like peptide-1 receptor agonist); HF (heart failure); HFmrEF (heart failure with mildly reduced ejection fraction); HFpEF (heart failure with preserved ejection fraction); HFrEF (heart failure with reduced ejection fraction); MASLD (metabolic dysfunction-associated steatotic Liver Disease); MRAs (mineralocorticoid receptor antagonists); SCORE2 (systematic coronary risk evaluation 2); SGLT2is (sodium–glucose cotransporter-2 inhibitors); TOD (target organ damage); T2DM (type 2 diabetes mellitus); UACR (urinary albumin-to-creatinine ratio).

Over the past five years, clinical trials have shown promising results for therapies such as GLP-1 receptor agonists, dual GIP/GLP-1 receptor agonists, SGLT2 inhibitors, and finerenone across the CRHM spectrum [55,56,57,58,59,60,61,62,63,64,65,66,67,68,69,70,71,72,73,74,75,76,77,78,79,80,81,82,83,84,85,86,87,88]. These agents target shared mechanisms—insulin resistance, inflammation, fluid retention, and neurohormonal imbalance—providing benefits that reach beyond single-organ conditions. As a result, traditional distinctions among cardiology, endocrinology, nephrology, and hepatology are giving way to a more integrated approach. By leveraging the pleiotropic effects of these medications, clinicians can streamline treatment and enhance outcomes for patients with overlapping cardiometabolic disorders. An overview of the novel and emerging therapeutic approaches for the CRHM syndrome along with the evidence from phase III clinical trials is presented in Table 3 and illustrated in Figure 3.

Many clinical trials are underway to further expand the indications of novel agents for the CRHM spectrum. Finerenone, already approved for diabetic CKD, is being investigated to determine its benefits in HF across the entire spectrum—including acute HF—as well as in non-diabetic CKD [71]. Meanwhile, the ESSENCE trial is evaluating novel therapies for MASH, with preliminary positive results reported but not yet published [86]. There is also a need for dedicated studies of GLP-1RAs in non-diabetic CKD. Moreover, promising data on GLP-1RAs and dual GIP/GLP-1RAs for HF and especially HFpEF from the SELECT, STEP-HFpEF, STEP-HFpEF DM, FLOW, and SUMMIT trials underscore the importance of additional research, particularly for HFrEF where evidence is scarce. Collectively, these efforts aim to expand treatment options and improve outcomes across the intertwined conditions that define the CRHM syndrome.

## 6. Implications for Clinical Practice and Patient Care

Despite advances in the understanding and management of CRHM diseases, traditional care models, which are often structured around individual organ systems or isolated specialties, fail to address the multifaceted and interconnected nature of the CRHM syndrome. This fragmented nature of current healthcare delivery results in siloed care, where patients are managed by multiple specialists—cardiologists, endocrinologists, nephrologists, hepatologists, and others—each focusing on a narrow aspect of the disease process. Consequently, patients frequently undergo redundant diagnostic tests, receive conflicting treatment recommendations, and are exposed to polypharmacy, increasing the risk of medication errors and adverse effects. This fragmented system can overwhelm patients, leading to confusion and poor adherence to treatment plans, perpetuating disease progression and poor outcomes.

To address these challenges, healthcare systems and professional societies must prioritize the establishment of integrated care models that dismantle traditional silos. These models should center around comprehensive, patient-focused clinics, uniting a multidisciplinary team of specialists, including cardiologists, endocrinologists, nephrologists, hepatologists, sleep specialists, exercise physiologists, dietitians, psychologists, and other allied health professionals. Such a coordinated framework ensures that all aspects of the CRHM syndrome are evaluated and managed collectively, fostering continuity of care and improved outcomes. Furthermore, the formation of guidelines for an integrated approach in the investigation and management of the CRHM syndrome can potentially unify its management across different specialties.

Equally essential is the formation of structured fellowships and subspecialty training programs dedicated to the management of CRHM diseases. By equipping healthcare professionals with the knowledge and skills to address the full CRHM, such initiatives can drive a shift from fragmented to integrated and continuous care.

## 7. Conclusions

The evolving concept of cardiometabolic disease underscores the deep interconnections among the heart, kidneys, liver, and metabolic pathways. While the CKM syndrome laid crucial groundwork for unifying these systems, the inclusion of the liver in the CRHM framework acknowledges the vital influence of MASLD and its progression to MASH on disease outcomes. By integrating hepatic dysfunction into clinical staging, diagnostics, and therapy selection, clinicians gain a more comprehensive view of patient risk and opportunities for intervention.

Novel therapeutic agents—such as SGLT2 inhibitors, GLP-1 receptor agonists, dual GIP/GLP-1 receptor agonists, and finerenone—demonstrate that targeting shared mechanisms can confer multi-organ protection. Recent trials indicate significant benefits in conditions spanning obesity, HF, CKD, and MASLD, blurring traditional boundaries among cardiology, nephrology, hepatology, and endocrinology. Ongoing research, including dedicated trials for non-diabetic CKD, HFrEF, and MASH, promises to refine the scope of these therapies, broaden their indications, and potentially transform management of patients with overlapping cardiometabolic disorders.

Despite these advances, care for patients with overlapping CRHM diseases remains highly fragmented, often siloed within individual specialties such as cardiology, nephrology, hepatology, and endocrinology. The proposed CRHM syndrome provides an opportunity to unify existing guidelines, develop new consensuses, and foster interdisciplinary collaboration among these fields. By emphasizing the interconnected nature of the heart, kidneys, liver, and metabolic pathways, CRHM syndrome could also pave the way for the establishment of novel fellowships or subspecialties dedicated to training clinicians along the entire CRHM spectrum, ensuring a new generation of specialists equipped to deliver holistic, patient-centered care.

## Figures and Tables

**Table 1:** The proposed staging system of the CRHM syndrome.

CRHM health stages	Definition
Stage 0: No CRHM risk factors	Individuals without overweight/obesity, abdominal obesity, metabolic risk factors (arterial hypertension, hypertriglyceridemia, MetS, diabetes mellitus), CKD, MASLD, or subclinical/clinical CVD
Stage I: Excess and/or dysfunctional adiposity	▪ Stage IIa: Excess adiposity ○ Stage IIa1: Overweight (BMI ≥25 kg/m^2^) or high-normal waist circumference (≥80/94 cm in women/men) ○ Stage IIa2: Obesity (BMI ≥30 kg/m^2^) or abdominal obesity (waist circumference ≥88/102 cm in women/men) ▪ Stage IIb: Dysfunctional adiposity ○ Excess adiposity with prediabetes (fasting blood glucose ≥100–125 mg/dL, HbA1c 5.7-6.4%, or OGTT with plasma glucose ≥140–199 mg/dL at 2 h)
Stage II: Metabolic risk factors, CKD, or MASLD	▪ Individuals with one or more of the following ○ Metabolic risk factors (arterial hypertension, hypertriglyceridemia, MetS, diabetes mellitus) ○ Low or moderate-risk CKD (according to KDIGO classification) ○ MASLD with fibrosis stage F0–F1
Stage III: Subclinical CVD	▪ Subclinical ASCVD or stage B HF among individuals with excess/dysfunctional adiposity, other metabolic risk factors, CKD, or MASLD ▪ Risk equivalents of subclinical CVD: ○ Very high 10-year cardiovascular risk (according to SCORE-2, SCORE-2OP, or SCORE-2 Diabetes) ○ High or very high-risk CKD (according to KDIGO classification) ○ MASLD with fibrosis stage F2–F4.
Stage IV: Clinical CVD	▪ Clinical CVD (ASCVD, HF) among individuals with excess/dysfunctional adiposity, other metabolic risk factors, CKD, or MASLD ○ Stage IVa: Without end-stage renal disease or cirrhosis. ○ Stage IVb: With end-stage renal disease (CKD stage V, under renal re-placement therapy, or kidney transplantation) and/or cirrhosis.

Abbreviations. ASCVD (atherosclerotic cardiovascular disease); BMI (body mass index); CKD (chronic kidney disease); CVD (cardiovascular disease); FPG (fasting plasma glucose); HbA1c (hemoglobin A1c); HF (heart failure); KDIGO (kidney disease: improving global outcomes); MASLD (metabolic dysfunction-associated steatotic liver disease); MetS (metabolic syndrome); OGTT (oral glucose tolerance test); SCORE-2 (systematic coronary risk evaluation for individuals under 70 years); SCORE-2 Diabetes (systematic coronary risk evaluation for individuals with diabetes); SCORE-2OP (systematic coronary risk evaluation for older people).

**Table 2:** Evaluation of the CRHM syndrome.

**Domain**	**Primary Assessments**	**Additional/Follow-up Tests**	**Rationale**
Adiposity	- Clinical evaluation of BMI, hip and waist circumference	- Bioimpedance analysis, DEXA, or underwater weighing for precise determination of fat mass and lean mass - If clinical suspicion exists, rule out endocrine causes (e.g., TSH for hypothyroidism, high-dose dexamethasone suppression test for Cushing’s) - Polysomnography if obstructive sleep apnea is clinically suspected	Identifies excess adiposity and addresses possible secondary hormonal factors contributing to obesity. A precise measure of body composition aids in tailoring lifestyle or medical interventions. Screening for OSA is crucial given its frequent association with obesity and cardiometabolic disorders.
Insulin Resistance & Diabetes Mellitus	- Fasting plasma glucose - Oral glucose tolerance test - Hemoglobin A1c - HOMA-IR	- Diabetes-related autoantibodies (if T1DM or LADA suspected) - Genetic tests (if MODY suspected)	Detects impaired glucose metabolism and clarifies the diabetes subtype, which is crucial for guiding appropriate therapy. Early diagnosis of insulin resistance helps prevent progression to overt diabetes and associated microvascular and macrovascular complications.
Arterial Hypertension	- Office and home blood pressure measurements	- Ambulatory blood pressure monitoring (if indicated) - Screening for secondary causes in resistant/atypical cases (e.g., plasma aldosterone/renin ratio, renal artery imaging)	- Essential for diagnosing and characterizing hypertension severity, a central driver of CRHM syndrome progression. - Identifying secondary causes ensures targeted therapy and improved blood pressure control, thereby reducing cardiovascular and renal risks.
Dyslipidemia and hyperuricemia	- Total cholesterol, LDL-C, HDL-C, Triglycerides, Lipoprotein(a) - Serum uric acid	- Advanced lipid testing (e.g., ApoA1, ApoB, lipoprotein particle size, cholesterol efflux capacity) or genetic testing for familial dyslipidemias if indicated	- Lipid profile abnormalities and hyperuricemia contribute to atherosclerosis, cardiovascular risk, and metabolic dysfunction. - Early identification and management of these conditions is critical for preventing long-term cardiovascular complications. - Particularly important to exclude heterozygous familial hypercholesterolemia in selected cases (e.g., family history of familial hypercholesterolemia, family history or early onset atherosclerosis, LDL-C >190)
CKD	Serum creatinine, eGFR, serum or urea nitrogen, urine ACR	- Renal ultrasound (if abnormalities detected) - Specialized tests for rarer causes (e.g., autoantibody workup, genetic testing in young-onset CKD)	- Identifies early CKD through proteinuria and declining eGFR. Early recognition and intervention can slow progression and help manage the renal component of CRHM syndrome, reducing cardiovascular morbidity.
MASLD	- Liver function tests (LFTs) - FIB-4 index	- Liver elastography (FibroScan) in high-risk FIB-4 index or selected cases - Additional imaging (e.g., MRI-PDFF) or biopsy (in selected cases)	Screens for hepatic steatosis and early fibrosis linked to metabolic dysfunction, essential for diagnosing MASLD/MASH and guiding therapeutic decisions. Detecting and staging MASLD enables targeted interventions to reduce liver-related morbidity.
Cardiovascular Assessment	- NT-proBNP and high-sensitive cardiac troponin T or I - Echocardiography (in those with abnormal biomarkers or clinical suspicion) - Risk scoring with validated tools (e.g., SCORE2, SCORE2-OP, SCORE2-Diabetes)	- Functional ischemic testing (e.g., stress ECG, stress echocardiography, myocardial perfusion imaging), CCTA and CAC score - Vascular imaging (triplex of carotid/vertebral arteries, aorta, lower-limb arteries) if clinical suspicion is high (e.g., intermittent claudication, diminished peripheral pulses, history of stroke or TIA, known CAD) - Inflammatory biomarkers (e.g., high sensitive C-reactive protein, interleukin-6) - In patients with heart failure screen for multiple hormonal deficiency syndrome (growth hormone, testosterone and/or triiodothyronine deficiency)	- Provides a standardized estimate of the 10-year risk of major adverse cardiovascular events across various populations, guiding intensity of preventive strategies. - Helps detect clinical or subclinical cardiovascular disorders that are frequently present in the CRHM syndrome.
Reproductive Hormonal Assessment	- Clinical evaluation for signs of PCOS (e.g., irregular menses, hyperandrogenism), male functional hypogonadism (e.g., low libido, testicular atrophy), or POI (e.g., irregular periods, low estrogen symptoms) - Serum hormone levels (e.g., LH, FSH, estradiol, total testosterone, free testosterone, SHBG, PRL)	- Pelvic ultrasound for PCOS - Testicular ultrasound if testicular pathology is suspected - Pituitary imaging (MRI) if hyperprolactinemia or central causes of hypogonadism are suspected - Karyotype analysis or genetic counseling in severe/early-onset cases	- Identifies endocrine or reproductive disturbances that may be closely linked with obesity and insulin resistance. - Conditions such as PCOS are associated with metabolic abnormalities, while male hypogonadism or POI may worsen or reflect systemic metabolic stress.

Abbreviations. ACR (albumin-to-creatinine ratio); ALT (alanine aminotransferase); ApoA (apolipoprotein A); ApoB (apolipoprotein B); AST (aspartate aminotransferase); BMI (body mass index); CAC (calcium score); CAD (coronary artery disease); CCTA (computed tomography angiography); CKD (chronic kidney disease); CRHM (cardio-renal-hepatic-metabolic); DEXA (dual-energy x-ray absorptiometry); eGFR (estimated glomerular filtration rate); FIB-4 (fibrosis-4 index); FSH (follicle-stimulating hormone); HDL-C (high-density lipoprotein cholesterol); HOMA-IR (homeostatic model assessment of insulin resistance); LDL-C (low-density lipoprotein cholesterol); LFTs (liver function tests); LH (luteinizing hormone); MASLD (metabolic-associated steatotic liver disease); MASH (metabolic-associated steatohepatitis); MRI (magnetic resonance imaging); MRI-PDFF (MRI proton density fat fraction); NT-proBNP (natriuretic peptide); OGTT (oral glucose tolerance test); PCOS (polycystic ovary syndrome); POI (primary ovarian insufficiency); PRL (prolactin); SCORE2 (systematic coronary risk evaluation 2); SHBG (sex hormone-binding globulin); TSH (thyroid-stimulating hormone).

**Table 3:** Novel and emerging therapeutic approaches for CRHM syndrome.

Condition	Emerging/Novel Therapies	Rationale/Evidence	References
Obesity	GLP-1RAs, GIP/GLP-1RAs	SCALE Program, STEP Program, SURMOUNT Program	[55,56,57,58,59,60]
HFpEF/HFmrEF	SGLT2i, GLP-1RAs, GIP/GLP-1RAs, finerenone	EMPEROR-Preserved, DELIVER, SELECT, STEP-HFpEF, FLOW, SUMMIT, FINE-ARTS, FINALITY-HF*	[61,62,63,64,65,66,67,68,69,70,71]
HFrEF	SGLT2i, GLP-1RAs, GIP/GLP-1RAs	Emperor-Reduced, DAPA-HF, FLOW, SELECT	[66,68,72,73]
Acute HF	SGLT2i	EMPULSE, SOLOIST-WHF, REDEFINE-HF*, CONFIRMATION-HF*	[71,74,75]
ASCVD	SGLT2i, GLP-1RAs	Meta-analyses of RCTs (for diabetic patients), SELECT (for non-diabetic patients)	[76,77,78]
Diabetic CKD	SGLT2i, GLP-1RAs, finerenone	Meta-analyses of RCTs, FLOW, FIGARO-DKD, FIDELIO-DKD, FIDELITY, EMPA-KIDNEY, DAPA-CKD, FINE-ONE*	[71,77,78,79,80,81,82]
Non-diabetic CKD	SGLT2i	EMPA-KIDNEY, DAPA-CKD, FIND-CKD*	[71,83,84]
MASLD	Resmetirom, GLP-1RAs, GIP/GLP-1RAs	MAESTRO-NASH, ESSENCE, SYNERGY-NASH	[85,86,87,88]

* Ongoing phase III trials are marked with an asterisk. Abbreviations. ASCVD (atherosclerotic cardiovascular disease); CKD (chronic kidney disease); GIP/GLP-1RA (glucose-dependent insulinotropic polypeptide and glucagon-like peptide-1 receptor agonist); GLP-1RA (glucagon-like peptide-1 receptor agonist); HF (heart failure); HFpEF (heart failure with preserved ejection fraction); HFmrEF (heart failure with mildly reduced ejection fraction); HFrEF (heart failure with reduced ejection fraction); MASLD (metabolic-associated steatotic liver disease); SGLT2i (sodium-glucose cotransporter-2 inhibitor).
